# Catalytic Soot Oxidation Activity of NiO–CeO_2_ Catalysts Prepared by a Coprecipitation Method: Influence of the Preparation pH on the Catalytic Performance

**DOI:** 10.3390/ma12203436

**Published:** 2019-10-21

**Authors:** Amar Bendieb Aberkane, María Pilar Yeste, Djazi Fayçal, Daniel Goma, Miguel Ángel Cauqui

**Affiliations:** 1 Laboratoire de recherche sur la physico-chimie des surfaces et interfaces (LRPCSI), Département de Pétrochimie & Génie des Procédés, Faculté de Technologie, Université 20 Août 1955-Skikda, BP 26, Route d’El Hadaiek-Skikda 21000, Algerie; f.djazi@univ-skikda.dz; 2 Department of Material Science, Metallurgical Engineering and Inorganic Chemistry, Faculty of Sciences, University of Cadiz, E-11510, Puerto Real, 11003 Cadiz, Spain; dani.gomajimenez@gm.uca.es (D.G.); miguelangel.cauqui@uca.es (M.Á.C.)

**Keywords:** nickel, cerium, coprecipitation, soot oxidation, mixed oxides

## Abstract

A series of NiO–CeO_2_ mixed oxide catalysts have been synthesized by a modified coprecipitation method at three different pH values (pH = 8, 9, and 10). The NiO–CeO_2_ mixed oxide samples were characterized by TGA, XRD, inductively coupled plasma atomic emission spectroscopy (ICP-AES), FTIR, Brunauer–Emmett–Teller (BET) surface area, H_2_ temperature-programmed reduction (H_2_-TPR), and electron microscopy (high-angle annular dark-field transmission electron microscopy/energy-dispersive X-ray spectroscopy (HAADF-TEM/EDS)). The catalytic activities of the samples for soot oxidation were investigated under loose and tight contact conditions. The catalysts exhibited a high BET surface area with average crystal sizes that varied with the pH values. Electron microscopy results showed the formation of small crystallites (~5 nm) of CeO_2_ supported on large plate-shaped particles of NiO (~20 nm thick). XRD showed that a proportion of the Ni^2+^ was incorporated into the ceria network, and it appeared that the amount on Ni^2+^ that replaced Ce^4+^ was higher when the synthesis of the mixed oxides was carried out at a lower pH. Among the synthesized catalysts, Ni-Ce-8 (pH = 8) exhibited the best catalytic performance.

## 1. Introduction

The fight against air pollution caused by the emission of pollutants from various sources is one of the main concerns for the international community. Soot particulates are formed as undesired by-products; in particular, these particles are emitted from incomplete internal combustion in engines together with NO_x_, CO, and hydrocarbons. These pollutants are widely produced by vehicles, ships, working machines, industries, etc. The removal of such particulates is of great importance due to the massive problems related to health and the environment [1,2,3]. The removal of these harmful particulates (soot particulate) is currently a huge task for researchers due to the increasing demands of environmental protection given the new environmental legislation regarding exhaust specifications [4,5]. The negative impact on nature caused by these kinds of pollutants has led researchers to develop new devices/innovations along with new synthetic methods that are more economical and non-polluting and that work under reasonable conditions to reduce soot emissions from engines. The ultimate aim is that new technologies will help to efficiently eliminate the problems related to soot-particulate emission [5,6]. Trapping of soot by filters (Gasoline Particulate Filters (GPFs) or Diesel Particulate Filters (DPFs) is a good way to decrease soot emission from exhaust gases. However, a periodic regeneration of these filters is necessary in order to avoid filter blocking or damage. During regeneration, the filter temperature is increased as the soot oxidizes at higher temperatures. The direct oxidation of this soot requires a high temperature (around 600–700 °C, depending on the operation conditions) and this in turn requires significant energy input [7,8]. The high temperature necessary for the regeneration of the uncatalyzed filter is generally achieved by injection of diesel/gasoline fuel into the exhaust system. Generally, the temperature range of the exhaust systems is around 600–800 °C for gasoline and 150–500 °C for diesel. This strategy leads to an uncontrolled exothermal phenomenon, which can damage the filter [8]. In the last few decades, significant improvements have been made by equipping exhaust systems with a catalytic converter. Noble metals (Pt, Pd, and Rh) are the most widely used catalysts due to their activity and durability. However, these noble metals are expensive, and their uses are limited in the catalytic soot oxidation reaction [7]. Therefore, the development of effective and active catalyst formulations that can significantly increase the rate of soot oxidation is of great interest [9,10].

Various types of catalysts that exhibit good catalytic activity for soot oxidation have been reported and these include metals and mixed metal oxides combined with noble metals [11,12,13,14], perovskite and perovskite-like oxides [15,16,17], transition metal oxides [18,19], rare earth modified catalysts [20,21], and noble metals [22,23,24]. Ceria and ceria-based catalysts are widely adopted for this purpose, and these systems have been studied and reported in several investigations on soot oxidation [1,5,25,26]. Such materials are considered as good catalysts for soot oxidation, mainly due to their high oxygen storage capacity and redox properties [1,21,27]. Ceria catalytic activity can be improved by the incorporation of different elements within the crystal lattice. It has been reported that the incorporation of Co, Fe, Cu, Zr, and Mn into ceria significantly modifies the oxygen storage capacity and can positively promote the soot oxidation activity [28,29,30]. Shan et al. studied the soot oxidation reaction over MnO_x_–CeO_2_ oxides and found that the catalytic activity depended on the preparation conditions, with the differences in catalytic activities attributed to the oxygen transfer capability [31]. Venkataswamy et al. reported nanostructured Ce_0.7_Mn_0.3_O_2–δ_ and Ce_0.7_ Fe_0.3_O_2–δ_ solid solutions for diesel soot oxidation and determined that Mn-doped CeO_2_ exhibited higher catalytic activity and better stability than the Fe-doped CeO_2_ sample [32]. Grabchenko et al. [33,34] reported that CeO_2_-based catalysts containing Ag within the CeO_2_ structure can greatly improve the reactivity of CeO_2_ toward soot oxidation. According to these reports, transition metals combined with ceria oxides can significantly affect the soot oxidation reaction by decreasing the onset temperature and the activation energy of the process.

The aim of the work described here was to study the effect of pH on the structure and properties of a series of NiO–CeO_2_ mixed oxides and to correlate the structure of the oxides with the catalytic activity in soot oxidation.

## 2. Materials and Methods 

### 2.1. Preparation of Catalysts

Ni–Ce mixed oxide catalysts with a calculated molar ratio Ni/Ce = 3 were synthesized by coprecipitation following the hydrotalcite route at different pH values of 8, 9, and 10 (± 0.22). Starting nitrates for M^(II)^ [Ni(NO_3_)_2_.6H_2_O, Sigma Aldrich, Schnelldorf, Germany, ≥98%] and M^(III)^ [Ce(NO_3_)_3_.6H_2_O, Sigma Aldrich, Schnelldorf, Germany, ≥99%] were dissolved in pure water with an appropriate molar ratio to form solution A. Na_2_CO_3_ (Cheminova International S.A, Madrid, Spain, ≥98%) was dissolved in pure water to form the alkaline solution B. For the synthesis, solution A was added dropwise to solution B under vigorous stirring at room temperature, while the pH value of the mixture was kept constant by using a 2 mol/L NaOH solution. The mixtures were stirred for 24 h at room temperature and the resulting precipitates were transferred to the reflux system and kept under vigorous stirring at 85 °C for 24 h. The resulting gels were washed with pure water and ethanol several times to remove any excess salts. After filtration and washing/centrifugation, the solids were dried overnight in an oven at 100 °C. The solids were then calcined in a muffle furnace at 600 °C for 1 h, with a heating ramp of 10 °C/min. The resulting materials were denoted as Ni-Ce-8, Ni-Ce-9, and Ni-Ce-10, where the numbers indicate the pH used in the synthesis.

### 2.2. Catalyst Characterization

The Ni-Ce-pH catalysts were characterized by several techniques before and after calcination at 600 °C. 

Thermogravimetric analysis (TGA) was carried out to identify the optimum calcination temperature. These experiments were performed in a thermogravimetric analyzer (TA instruments Q50) (TA instruments, New Castle, DE, USA). Fresh samples were introduced into an alumina microbalance pan and heated up to 900 °C with a temperature ramp of 10 °C/min under an air/nitrogen mixture (60/40 v/v, with a total flow of 100 mL/min).

In order to identify the surface functional groups on the prepared catalysts, Fourier-transform infrared spectroscopy (FTIR) was carried out on a Bruker Alpha and/or Nicolet iS10 spectrophotometer (Bruker Optik GMBH, Ettlingen, Germany) at high resolution and over 32 scans to improve the signal-to-noise ratio in the wavelength range between 4000 and 400 cm^−1^. Self-supporting pressed pellets of the neat powders and/or KBr powder mixtures were used (3% of dried powder was mixed and ground in a mortar with 97% KBr).

Inductively coupled plasma atomic emission spectroscopy (ICP-AES) (thermo elemental IRIS Intrepid) (Thermo Scientific, Waltham, MA, USA) was used to determine the chemical composition of the prepared catalysts. 

Powder XRD patterns were recorded on a Bruker D8 Advance A-25 diffractometer (Bruker Corporation, Billerica, MA, USA) working in Bragg–Brentano geometry using Cu-K_α_ radiation (λ = 1.54 Å) as the radiation source. X-ray diffractograms were collected at room temperature over the 2 theta range from 10° to 80°, with a stepwise increment of 0.02° and an acquisition time of 3 s at every angle. After the data collection, the XRD patterns were indexed in accordance with the reference patterns available in the Joint Committee on Powder Diffraction Standards, JCPDS database. Scherrer (Equation (1)) and Bragg (Equation (2)) equations were used for the crystallite size and lattice parameters calculation of the catalysts.

(1) $$L=\frac{0.9\lambda_{k\alpha 1}}{B_{\left(2\theta \right)}{\cos\;\theta }_{\max}},$$

where L denotes the average particle size, 0.9 is the value in radians when B_(2θ)_ is the full-width at half maximum (FWHM) of the peak, λ_kα1_ is the wavelength of the X-ray radiation (0.15406 nm), and θ_max_ is the angular position at the (111) peak maximum of the oxide NiO or CeO_2_.

(2) $$2d_{\mathrm{hkl}}\mathrm{Sin}\left(\theta \right)=n\lambda ,$$

where d_hkl_ is the spacing of the crystal layers (path difference), λ is the wavelength of the X-ray, θ is the incident angle (the angle between incident ray and the scatter plane) and n is an integer. Then, knowing d_hkl_ from Equation (2), we can estimate the lattice parameter **a** from Equation (3):

(3) $$d_{\mathrm{hkl}}=\frac{a}{\sqrt{\left(h^{2}+k^{2}+l^{2}\right)}}.$$

The textural properties of the catalysts were determined by means of N_2_ adsorption–desorption isotherms at −196 °C using an automatic volumetric system (Autosorb iQ3, Quantachrome Instruments, Boynton Beach, FL, USA). Prior to the measurement, the samples were degassed under vacuum for 2 h at 200 °C to remove physically adsorbed components and other adsorbed gases from the catalyst surface. The specific surface areas (S_BET_) of the catalysts were calculated from the multipoint BET (Brunauer–Emmett–Teller) method. The pore volume of the catalysts was obtained using the BJH method (Barrett–Joyner–Halenda).

The reducibility of the catalysts was investigated by H_2_ temperature-programmed reduction (H_2_-TPR) experiments. These experiments were carried out on a Micromeritics AutoChem II 2920 instrument (Norcross, GA, USA) automated characterization system equipped with a thermal conductivity detector (TCD) (Norcross, GA, USA). In a typical H_2_-TPR run, 50 mg of sample was treated under a 5% H_2_/Ar flow (50 mL/min) in the temperature range 30–900 °C at a heating rate of 10 °C/min. The hydrogen consumption was quantitatively determined after calibration of the TCD response using CuO as standard.

The morphologies of the catalysts were investigated by the high-angle annular dark-field scanning–transmission electron microscopy (HAADF-STEM) technique. The corresponding observations were performed using a JEOL 2010 F microscope (JEOL, Peabody, MA, USA), working at 200 kV, with a structural resolution of 0.19 nm. The digital diffractograms (DDPs) reported here correspond to the log-scaled power spectrum of the corresponding fast Fourier transforms. 

### 2.3. Catalytic Activity Measurement

Printex-U (Orion Engineered Carbons, Frankfurt, Germany) (from Degussa S.A.), the characterization of which is reported elsewhere [35], was used as a model soot, since its properties are similar to those of soot particulates. The catalytic activity was measured in both loose contact (mixed with a spatula for 5 min) and tight contact (ground in a mortar for 5 min) modes with catalyst–soot mixtures of 20:1 (w/w) [21]. The catalytic measurements were performed by thermogravimetric analysis (TA Instruments—WATERS LLC-Q50, TA Instruments, New Castle, UK) of the mixtures, heating from room temperature up to 800 °C, under a 60 mL/min flow of air balanced with N_2_ (total flow of 100 mL/min) flow.

The apparent activation energy of the soot oxidation was determined by the Ozawa method (Equation (4)) on the basis of TGA data. Ea represents the activation energy expressed in KJ/mol, and R represents the ideal gas constant expressed in KJ/(mol K). Each TGA profile provided the temperatures, T_x_, at which an ‘x%’ fixed fraction of soot conversion was obtained. Experiments were performed by varying the heating rate β (β = 10, 15, 20, and 25 °C/min).

(4) $$\frac{d\left(\log\left(\beta \right)\right)}{d\left(\frac{1}{T_{x}}\right)}=0.4567\frac{E_{a}}{R}.$$

## 3. Results and Discussion

This section is divided into subsections and it provides a concise and precise description of the experimental results, their interpretation, and the conclusions that can be drawn.

### 3.1. Catalyst Characterization

#### 3.1.1. Thermogravimetric Analysis (TGA) and Fourier-Transform Infrared Spectroscopy Analysis (FTIR)

The decomposition of Ni-Ce-pH (pH = 8, 9, and 10) precursors was studied by TGA from room temperature to 900 °C under a flow of air at atmospheric pressure. The TGA trace for each catalyst was recorded (Supplementary Materials, Figure S1). In general, the thermal evolution of the catalysts Ni-Ce-pH involved three main mass loss steps during the decomposition up to 600 °C. Above 600 °C, a weight loss was not observed. This finding indicates that a phase change did not occur after 600 °C. Therefore, the TGA results for our precursors show that 600 °C is the most appropriate temperature for calcination because it is high enough for the removal of water, carbonates, and nitrates.

**Figure 1:**
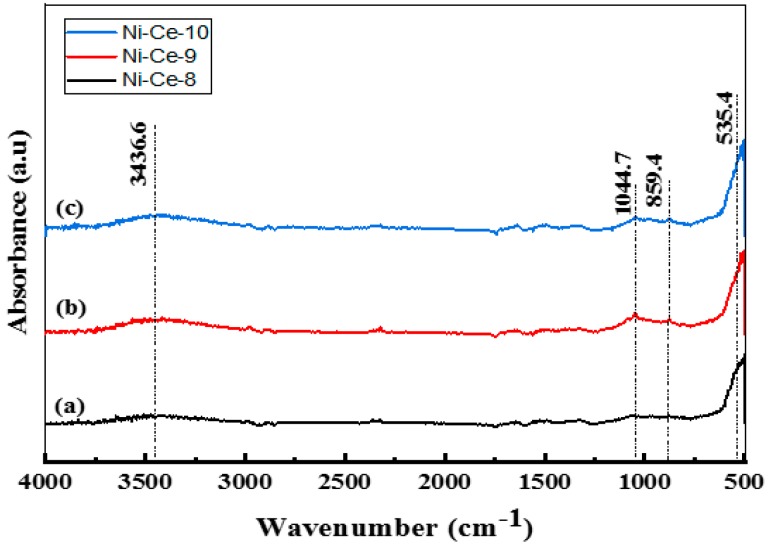
FTIR Spectra of Ni-Ce-pH catalysts after calcination at 600 °C: (**a**) Ni-Ce-8; (**b**) Ni-Ce-9 and (**c**) Ni-Ce-10.

After calcination at 600 °C, an FTIR study was carried out on the samples (Figure 1). In the FTIR spectra, the same bands approximately were observed as in the non-calcined catalysts (Figure S2), albeit with a weaker absorbance. The bands post-calcination diminished due to the removal of carbonates, nitrates, and water molecules from the catalyst structure. The presence of a band with a weak absorbance at 3436 cm^−1^ is attributed to the stretching vibration mode of the hydroxyl groups (–OH) of water, which was not completely removed. For the calcined systems, the presence of low frequency bands at 1044, 859, and 535 cm^−1^ can be attributed to (M–O) and (M–O–M) vibrations. The weak absorbance and diminished intensities of the bands indicate that calcination at 600 °C led to complete conversion of the structure of the catalyst to metal oxides.

#### 3.1.2. Elemental Composition (ICP)

The chemical composition of the mixed oxides was determined by ICP-AES (Table 1). The Ni/Ce ratio varied from 0.87 to 1, which is slightly lower than the nominal value (1.25) and suggests a loss of Ni during the synthetic procedure, especially when using pH = 8. The Ni/Ce ratio depends on the efficient precipitation of Ni and Ce particles under an optimal pH value. In our case, the Ni/Ce ratio was not particularly close to the nominal value because the Ni particles were not precipitated efficiently at the lower pH value. 

#### 3.1.3. X-Ray Diffraction Analysis (XRD)

**Figure 2:**
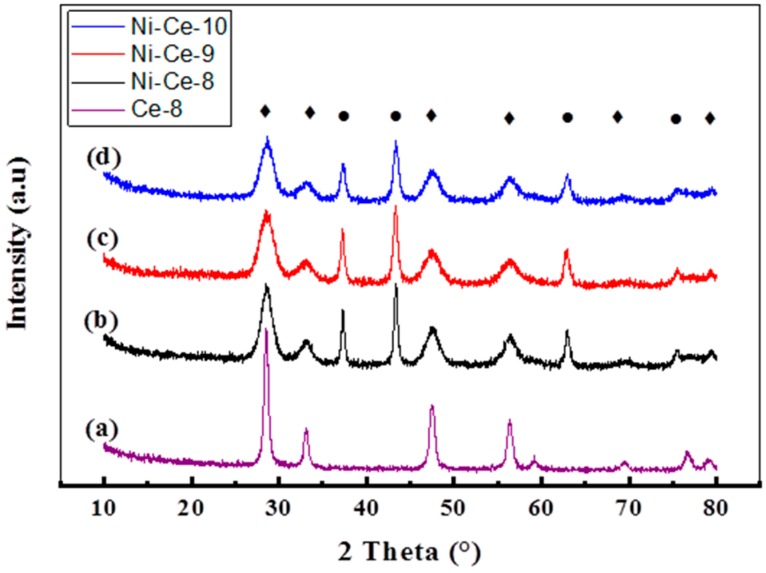
XRD patterns of Ni-Ce-pH catalysts calcined at 600 °C: (**a**) Ce-8; (**b**) Ni-Ce-8; (**c**) Ni-Ce-9; and (**d**) Ni-Ce-10. ♦ CeO_2_, ● NiO.

The X-ray diffractograms of Ni-Ce-pH catalysts calcined at 600 °C are shown in Figure 2. The XRD patterns are very similar and they show the presence of two series of peaks corresponding to the fluorite-like structure of CeO_2_ (reflections at 28.53°, 33.08°, 47.48°, 56.37°, 69.41°, and 79.2°, according to JCPDS 34-0394, JCPDS 65-2975) and to the cubic NiO phase (reflections at 37.3°, 43.36°, 62.94°, and 75.43°, according JCPDS 44-1159, PDF 47-1049). Values for the crystallite sizes and lattice parameters were estimated by using the Scherrer and Bragg equations (Table 2). As a reference, the XRD of a CeO_2_ prepared at pH = 8 using the same method is included. The calculated lattice parameters were close to those reported for both standard cubic NiO (4.1771 Å) [36], CeO_2_ (5.4110 Å) [3], and Ce-8 (5.410 Å). However, it is worth noting that, in the case of CeO_2_, the values obtained are always below the reported lattice parameter and the difference is higher in the case of the sample prepared at a lower pH (Ni-Ce-8). This effect has been observed by several authors and was explained as being due to the incorporation of Ni^2+^ into the ceria network [36,37]. According to these results, it seems that the amount on Ni^2+^ that replaces Ce^4+^ is higher when the synthesis of the mixed oxides is carried out at a lower pH.

The calculated crystallite sizes of NiO and CeO_2_ phases are also included in Table 2. In the case of CeO_2,_ except for Ce-8 used as a reference, average values of around 4–5 nm were obtained. The crystallites of NiO are larger and are also much more influenced by the pH. As deduced from the results in Table 2, the crystallite size for NiO decreased on increasing the pH.

#### 3.1.4. Surface Area BET Measurement

**Figure 3:**
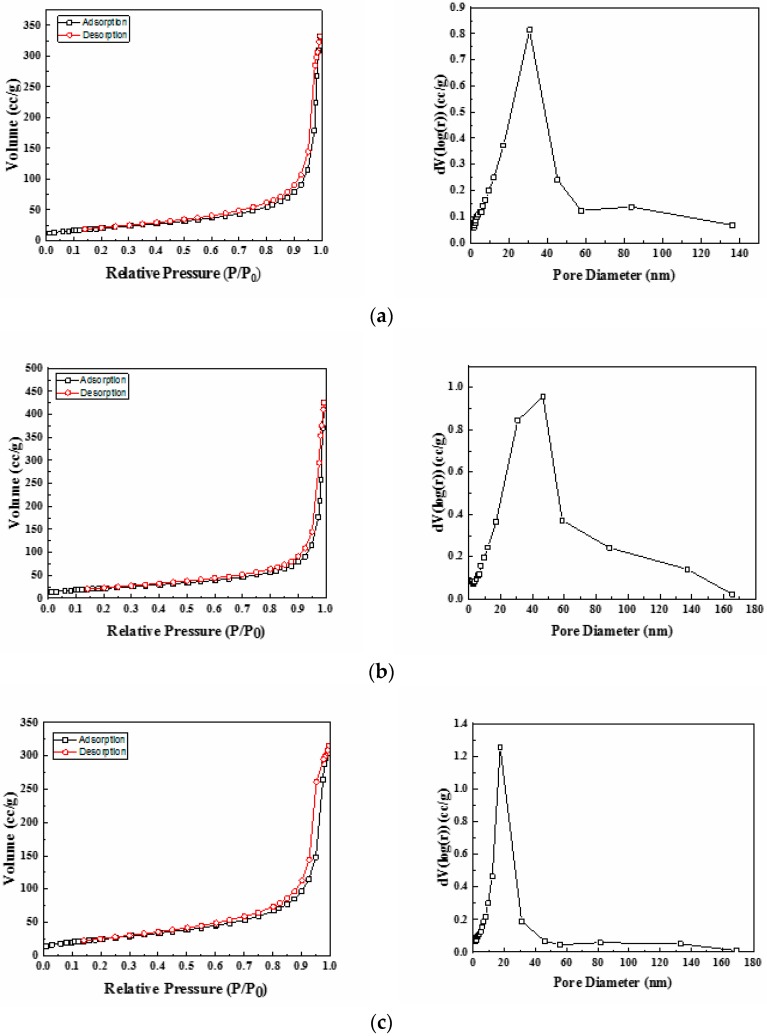
Nitrogen adsorption/desorption isotherms and pore size distribution of Ni-Ce-pH catalysts after calcination at 600 °C: (**a**) Ni-Ce-8; (**b**) Ni-Ce-9 and (**c**) Ni-Ce-10.

The textural properties of the synthesized Ni-Ce-pH (pH = 8, 9, and 10) mixed oxides were investigated by nitrogen adsorption–desorption isotherm measurements. The isotherms and the corresponding pore size distribution curves are depicted in Figure 3. According to the International Union of Pure and Applied Chemistry (IUPCA) classification, these catalysts displayed type IV nitrogen adsorption–desorption isotherms, which are typical of materials that have a mesoporous structure [38]. The values for average pore sizes obtained by applying the BJH method are in the range 20–80 nm (Table 3). Even at high P/P_0_ values, the N_2_ adsorption–desorption curves did not show a plateau, which suggests that nitrogen physisorption occurred between aggregates or agglomerates of particles that form slit-shaped pores with non-uniform size and/or shape [39]. Furthermore, it can be seen from Figure 3 that the pore size distribution curves only have one large uniform and intense peak, thus indicating a rather good uniformity of the pore channels in the mesoporous texture of these catalysts.

The BET specific surface area values of the catalysts are summarized in Table 3. The results show that on increasing the preparation pH the specific surface area increases significantly from 73 m^2^/g (pH = 8) to 91 m^2^/g (pH = 10), which is in good agreement with the changes in crystallinity observed by XRD for both CeO_2_ and NiO_2_ phases. 

#### 3.1.5. Temperature-Programmed Reduction (H_2_-TPR)

**Figure 4:**
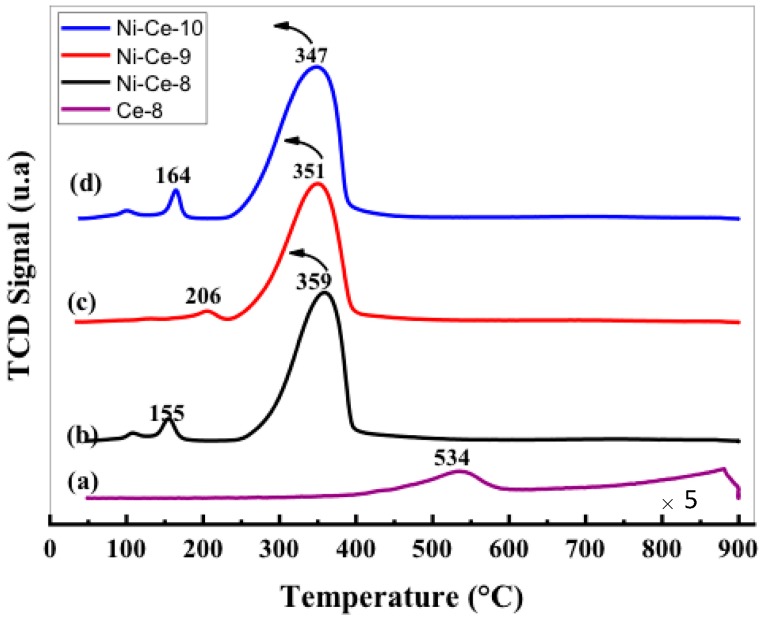
H_2_ temperature-programmed reduction (H_2_-TPR) profiles of Ni-Ce-pH catalysts after calcination at 600 °C: (**a**) Ce-8; (**b**) Ni-Ce-8; (**c**) Ni-Ce-9; and (**d**) Ni-Ce-10.

The reducibility of a catalyst is an important parameter that is very often associated with its activity in the soot oxidation reaction. The reducibility of our Ni–Ce mixed oxides was investigated by temperature-programmed reduction experiments under a flow of H_2_ (H_2_-TPR). The H_2_-TPR profiles are depicted in Figure 4. As a reference, the reduction of Ce-8 starts to be noticeable at temperature above 400 °C, and peaking at 534 and 870 °C. The introduction of nickel results in an oxide with improved reducibility. It can be seen that all of the profiles show a main asymmetric reduction peak in the range 250–400 °C along with some peaks with low intensity at temperatures around 150–200 °C. It is important to note that other hydrogen consumption signals were not observed at higher temperatures (>400 °C), which implies that all of the reduction processes occurring in these samples, including the reduction of CeO_2_, take place at a moderate temperature. For a better assignment of the TPR peaks, the H_2_-consumption values obtained experimentally from the integration of the curves were compared with the theoretical values estimated for complete reduction of the samples, assuming that, after calcination of the samples, all of the Ni is present as Ni^2+^ and Ce as Ce^4+^ (Table 4). According to these values, we conclude that the catalyst reduction is almost complete at the end of the TPR experiments, which indicates that the main reduction peak in the range 250–400 °C accounts for the reduction of both NiO (NiO→Ni) and CeO_2_ (CeO_2_→Ce_2_O_3_) components.

The position of this peak in the case of NiO catalysts is normally associated in the literature [40,41] with the NiO particle size (larger particles are reduced at higher temperatures) and/or with the degree of NiO-support interaction (stronger interactions lead to higher reduction temperatures). In this case, only a very small shift to lower temperatures was observed in the position of the maximum on increasing the pH. This effect could be explained in terms of the decrease in crystallite sizes with pH, observed by XRD. However, a difference in the interaction degree between NiO and CeO_2_ as a function of the synthetic conditions cannot be ruled out.

The peaks observed at low temperatures can be explained as being due to the reduction of oxygen adsorbed on the vacancies caused by the incorporation of Ni^2+^ within the CeO_2_ network. In this sense, it seems that the formation of these vacancies is especially favored in the cases of Ni-Ce-8 and Ni-Ce-10 [40,42].

#### 3.1.6. Electron Microscopy Analysis (HAADF/TEM-EDS)

**Figure 5:**
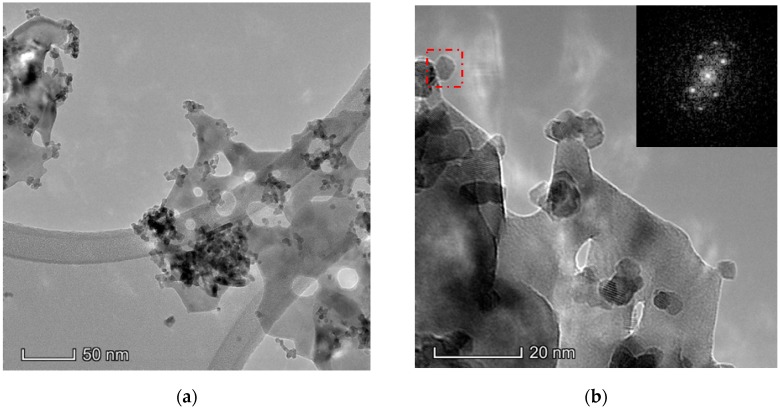
Representative TEM images of the Ni-Ce-8 catalyst: (**a**) low magnification; (**b**) high magnification; and as insets the digital diffractograms (DDPs) of the zone marked.

**Figure 6:**
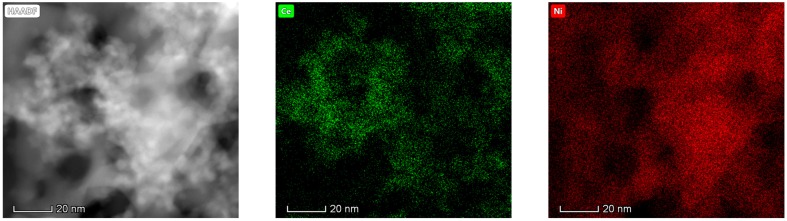

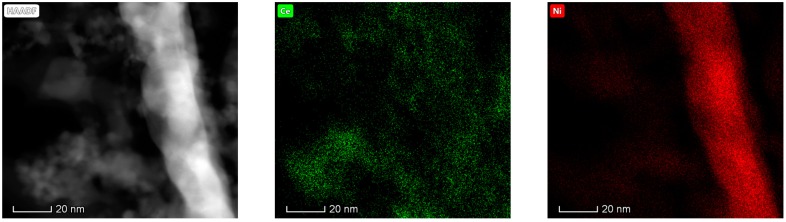
High-angle annular dark-field scanning–transmission electron microscopy (HAADF-STEM) images of Ni-Ce-8 and energy-dispersive X-ray spectroscopy (EDS) chemical maps of Ni and Ce.

**Figure 7:**
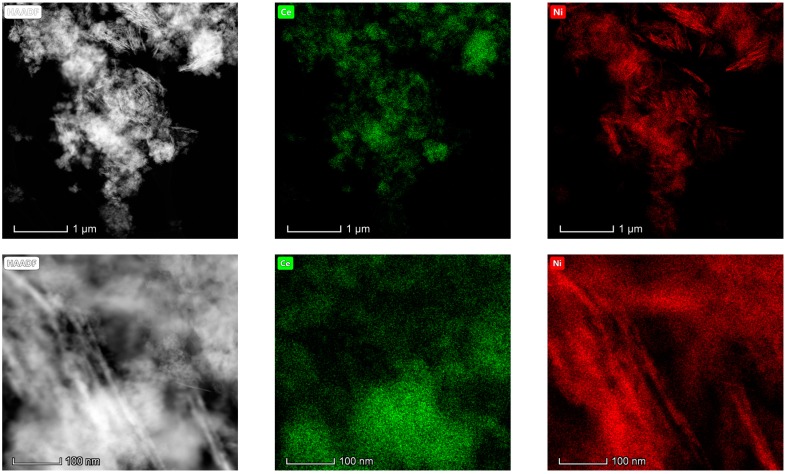
HAADF-STEM images of Ni-Ce-9 and EDS chemical maps of Ni and Ce.

**Figure 8:**
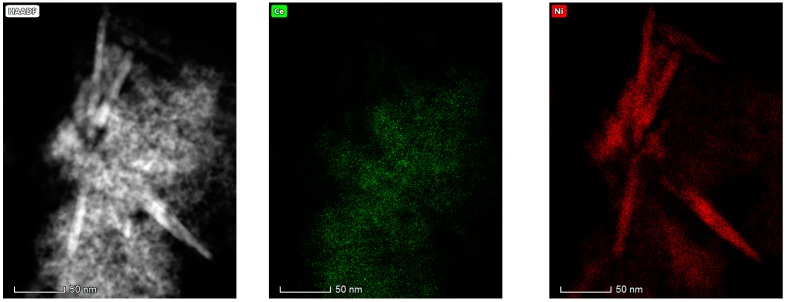
HAADF-STEM images of Ni-Ce-10 and EDS chemical maps of Ni and Ce.

The results obtained in the electron microscopy characterization of the Ni-Ce-X mixed oxides are presented in Figure 5, Figure 6, Figure 7 and Figure 8. A representative image of these samples taken at two different magnifications is shown in Figure 5. NiO was identified in the form of characteristic holey plate-shaped particles. In contrast, CeO_2_ appears to form small crystals with different degrees of agglomeration (the spots observed in the DDPs shown as insets were indexed on the basis of the fluorite structure). Some of these crystals can be observed in Figure 5, and they are isolated and have a size of around 5 nm, which is consistent with the results obtained by XRD. In an effort to gain more insight into the spatial distribution of Ni and Ce in the mixed oxides, STEM-EDS mappings of Ni and Ce were registered for the three catalysts. HAADF-STEM images of the analyzed area and the corresponding chemical maps are shown in Figure 6, Figure 7 and Figure 8. The HAADF-STEM image corresponding to the Ni-Ce-8 sample (Figure 6) clearly illustrates the structure of this catalyst, which is constituted by small crystallites supported on plate-shaped particles. The chemical maps confirm the presence of Ce in the crystallites and Ni in the plate-shaped particles. The image shown in Figure 6 corresponds to one of these plate-shaped particles in a profile view, which allows us to establish a thickness of around 20 nm for these particles. The same structure can be observed in the images corresponding to the Ni-Ce-9 and Ni-Ce-10. However, in the case of the Ni-Ce-9 sample, an additional feature can be highlighted, namely a higher population of stacked particles—as observed mainly in the low magnification HAADF-STEM image in Figure 7. In summary, the results obtained by electron microscopy indicate that the investigated Ni–Ce catalysts are constituted by two different morphologies: CeO_2_ forms small crystallites (~5 nm) supported on large plate-shaped particles of NiO (~20 nm thick). 

### 3.2. Catalytic Activity Tests

#### 3.2.1. Soot Oxidation under Air

**Figure 9:**
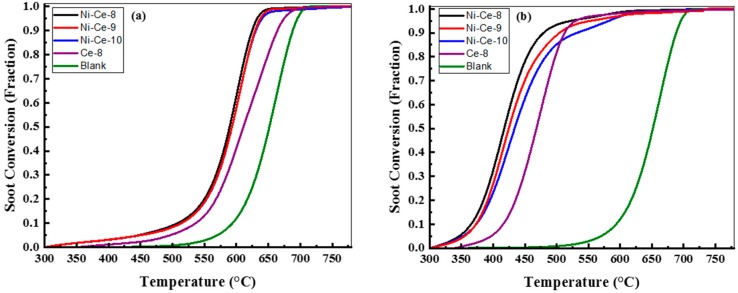
Soot oxidation with air/N_2_ over Ce-8, Ni-Ce-8, Ni-Ce-9, and Ni-Ce-10 catalysts as indicated, conditions: TGA, catalyst:soot—20:1 (w/w), contact: (**a**) loose and (**b**) tight, heating rate = 10 °C/min, air/N_2_ flow = 100 mL/min.

The samples of Ni-Ce-pH were tested in the catalytic soot oxidation reaction. As a reference, the results of a CeO_2_ prepared at pH = 8 using the same method is included. The catalytic activity measurements were made in two contact modes: loose (mixed with a spatula for 5 min) and tight (ground in a mortar for 5 min) conditions because the contact mode in this reaction is a key factor [43]. All TGA profiles, as a function of temperature of the Ce-8, Ni-Ce-8, Ni-Ce-9, Ni-Ce-10, and uncatalyzed soot, were normalized by removing the weight loss/soot conversion (below 300 °C) due to the desorption of adsorbed H_2_O [20]. The normalized soot conversions due to soot oxidation for both contact modes (loose and tight) of Ce-8 and Ni-Ce-pH mixed oxides are represented in Figure 9, and the results are summarized in Table 5. In order to facilitate a comparison, the combustion of soot referred to the uncatalyzed soot oxidation was also studied. The soot combustion for the uncatalyzed soot oxidation was completed between 590 and 705 °C. 

In the case of loose contact, Figure 9a, the catalytic activities of these catalysts are similar and better than that of Ce-8. In previous studies it has been reported that the influence of the catalysts in the loose contact mode is very limited [20,44,45,46]. However, according to [10,46], oxygen mobility through the catalysts with soot particulates plays an important role in the loose contact mode. In this case, these results were quite similar to each other because the redox properties were quite similar according to H_2_-TPR analysis. This would explain Ce-8 having the poorest activity due to the worst reducibility.

The soot conversions of soot mixed with the Ce-8, Ni-Ce-8, Ni-Ce-9, and Ni-Ce-10 catalysts under tight contact mode are provided in Figure 9b. Generally, in tight contact mode the grinding force achieved on using the mortar to prepare the catalyst–soot mixture generates a more effective contact between the catalyst and the sample [3]. The estimated T_50_ temperatures for the soot oxidation of the studied catalysts Ce-8, Ni-Ce-8, Ni-Ce-9, and Ni-Ce-10 were 469, 417, 424, and 433 °C, respectively. These results for soot oxidation with Ce catalysts are better than others found in the literature as obtained, for example, by Rangaswamy et al. [21], Shan et al. [31], and Krishna et al. [20]. In this case, the Ni particles played an important role in diminishing the T_50_ and increasing the catalytic activity.

It was observed that there was a high dependence between pH in the preparation step and catalytic activity. It was found that the highest conversion values were achieved for the sample prepared with the most acidic medium.

In an attempt to explain these differences, Wen et al. [47] reported that the surface area and mesoporous structure of the materials plays an important role in enhancing the contact between the soot and the active sites of the catalyst. However, in our study, the lower preparation pH and surface area gave rise to the highest activity. According to the literature [10,46], another factor that predominates in tight contact is the quantity of active oxygen species on the surface. In nickel and cerium mixed oxides, it is possible to create very reactive oxygen species because of the synergistic effect caused by Ni incorporated within the CeO_2_ [48,49]. As a consequence, it seems reasonable to envisage that Ni-Ce-8 has the highest catalytic activity because of the incorporation of nickel in the ceria network, as confirmed by XRD.

#### 3.2.2. Apparent Activation Energy

The apparent activation energy is an important parameter in soot oxidation reactions. Ozawa plots at different soot conversion levels over Ni-Ce-8, Ni-Ce-9, and Ni-Ce-10 mixed oxide catalysts are shown in Figure S3. The soot oxidation experiments using tight contact mode were carried out at different heating rates (β = 10, 15, 20, and 25 °C/min) for all catalysts in the TGA system. The activation energy (E_a_) can be estimated from the slope of the least-squares straight line fit of log(β) versus $1/T_{x}$ from Ozawa plots at the various soot conversion levels (x = 10%, 20%, 30%, 40%, 50%, 60%, and 70%). The activation energies of all catalysts are summarized in Table 6. It has been observed that the activation energy of non-catalytic soot at T_50_ (50% soot conversion) and as reported by Neeft et al. [50] was higher compared to that of the catalyzed one as mentioned in Table 6. The good interaction of Ni particles incorporated into ceria network may have a highly positive effect on the activation energy. It can be observed from the results in Table 6 that the activation energy is lower for catalysts prepared at pH 8. At low conversion levels (x = 10%, 20%, 30%, and 40%) the activation energy is lower for the catalyst prepared at pH 10 than for the catalyst prepared at pH 9. However, at high conversion levels, the activation energy increases with increasing pH. This finding could be related with the H_2_-TPR measurements, where in Ni-Ce-8 and Ni-Ce-10 at low temperature there is oxygen adsorbed in the vacancies caused by the incorporation of Ni^2+^ within the CeO_2_ network. 

## 4. Conclusions

Ni-Ce-pH (pH = 8, 9, and 10) mixed oxide catalysts have been successfully prepared by a modified coprecipitation method at different pH values. The catalytic activity of these catalysts was investigated for soot oxidation under loose and tight contact conditions using the TGA technique. Ni-Ce-pH mixed oxide catalysts prepared at different pH values exhibit different physicochemical properties that can have a marked influence on their catalytic activity in the soot oxidation reaction. Under tight contact mode, it was found that the catalytic activity increased with decreasing pH, and Ni-Ce-8 exhibited a better performance in soot combustion due to the high incorporation of nickel within the ceria network. However, under loose contact mode, the catalytic activities of these catalysts are very similar because this process is governed by bulk diffusion of lattice oxygen.

## Figures and Tables

**Table 1:** Chemical composition of Ni-Ce-pH catalysts.

Sample	Ni/Ce ^a^	Weight (%)		Ni/Ce ^b^
		Ce (wt %)	Ni (wt %)	
Ni-Ce-8	1.25	32.3 ± 0.1	28.0 ± 0.2	0.87
Ni-Ce-9	1.25	28.0 ± 0.3	28.0 ± 0.3	1
Ni-Ce-10	1.25	32.4 ± 0.2	32.2 ± 0.2	0.99

a Nominal value; b Measured value (ICP—inductively coupled plasma).

**Table 2:** Results obtained from the structural characterization of Ni–Ce catalysts by XRD.

Sample	Crystallite	Position of the Most Intense Peak 2θ (°)	Average Crystallite Size (nm)	Lattice Parameter (a) NiO and CeO2 (Å)
Ce-8	CeO_2_	28.56	12.6	5.410
Ni-Ce-8	NiO	43.36	18.7	4.170
	CeO_2_	28.54	5.2	5.397
Ni-Ce-9	NiO	43.32	14.3	4.173
	CeO_2_	28.53	4.2	5.399
Ni-Ce-10	NiO	43.37	13.0	4.169
	CeO_2_	28.56	4.8	5.405

**Table 3:** Textural properties of Ni-Ce-pH catalysts.

Sample	S_BET_ (m^2^/g)	V_P_ (cm^3^/g)	D_P_ (nm)
Ni-Ce-8	73	0.27	19.14
Ni-Ce-9	80	0.27	56.99
Ni-Ce-10	91	0.40	17.08

**Table 4:** H_2_ uptake in the H_2_-TPR analysis of the prepared catalysts.

Sample	Total H_2_ Consumption (mmol/g) ^(a)^	Total H_2_ Consumption (mmol/g) ^(b)^	ɛ ^(c)^
Ce-8	2.9	1.8	-
Ni-Ce-8	11.84	9.90	0.84
Ni-Ce-9	11.53	9.82	0.85
Ni-Ce-10	13.28	11.03	0.83

(a) Amount of H_2_ required for complete reduction (theoretical); (b) Amount of H_2_ consumed (experimental); (c) Extent of reduction calculated from the ratio between experimental and theoretical H_2_ consumption.

**Table 5:** Characteristic temperatures of soot oxidation in air in loose and tight contact mode for the prepared catalysts.

Contact Mode	Catalyst	Temperature (°C)			
		T_10_^a^	T_50_^a^	T_90_^a^	T_f_^b^
Uncatalyzed	Pure soot (Printex-U)	593	652	688	705
Loose	Ce-8	536	610	664	690
	Ni-Ce-8	507	589	624	646
	Ni-Ce-9	511	592	629	651
	Ni-Ce-10	513	594	630	655
Tight	Ce-8	415	468	537	617
	Ni-Ce-8	365	417	479	598
	Ni-Ce-9	372	424	504	611
	Ni-Ce-10	375	433	531	616

a Temperature at 10%, 50%, and 90% soot conversion; b Final temperature of soot oxidation.

**Table 6:** Apparent activation energy (E_a_) calculated from Ozawa plots at different soot conversions. The activation energy of non-catalytic soot at T_50_ is 168 KJ/mol.

Catalyst	Apparent Activation Energy (kJ/mol) ± 5, at x% Conversion						
	10	20	30	40	50	60	70
Ni-Ce-8	109	111	114	118	121	126	133
Ni-Ce-9	139	144	140	139	130	141	142
Ni-Ce-10	133	125	128	131	136	142	150

## Supplementary Materials

The following are available online at https://www.mdpi.com/1996-1944/12/20/3436/s1, Figure S1: TGA profile of Ni-Ce-pH catalysts: (a) Ni-Ce-8, (b) Ni-Ce-9, and (c) Ni-Ce-10; Figure S2: FTIR spectra of Ni-Ce-pH catalysts before calcination: (a) Ni-Ce-8, (b) Ni-Ce-9, and (c) Ni-Ce-10; Figure S3: Ozawa plots over (a) Ni-Ce-8, (b) Ni-Ce-9, and (c) Ni-Ce-10 at different soot conversion levels (x%), with (β) different heating applied during the soot oxidation, and T_x_ (temperature at ‘x%’ conversion).

## References

[1] Bueno-López A. (2014). Diesel soot combustion ceria catalysts. Appl. Catal. B Environ..

[2] Liu J., Zhao Z., Chen Y., Xu C., Duan A., Jiang G. (2011). Different valent ions-doped cerium oxides and their catalytic performances for soot oxidation. Catal. Today.

[3] Zhang W., Niu X., Chen L., Yuan F., Zhu Y. (2016). Soot Combustion over Nanostructured Ceria with Different Morphologies. Sci. Rep..

[4] Xiao P., Zhong L., Zhu J., Hong J., Li J., Li H., Zhu Y. (2015). CO and soot oxidation over macroporous perovskite LaFeO_3_. Catal. Today.

[5] Simonsen S.B., Dahl S., Johnson E., Helveg S. (2008). Ceria-catalyzed Soot Oxidation studied by Environmental Transmission Electron Microscopy. J. Catal..

[6] Cousin R., Capelle S., Courcot D., Aboukaı A. (2007). Copper-vanadium-cerium oxide catalysts for carbon black oxidation. Appl. Catal. B Environ..

[7] Neelapala S.D., Dasari H. (2018). Catalytic soot oxidation activity of Cr-doped Ceria (Ce_1−x_Cr_x_O_2−δ_) synthesized by sol-gel method with organic additives. Mater. Sci. Eng. Technol..

[8] Shimizu K., Kawachi H., Satsuma A. (2010). Study of active sites and mechanism for soot oxidation by silver-loaded ceria catalyst. Appl. Catal. B Environ..

[9] Obeid E., Lizarraga L., Tsampas M.N., Cordier A., Boréave A., Steil M.C., Blanchard G., Pajot K., Vernoux P. (2014). Continuously regenerating Diesel Particulate Filters based on ionically conducting ceramics. J. Catal..

[10] Aneggi E., De Leitenburg C., Trovarelli A. (2012). On the role of lattice/surface oxygen in ceria—Zirconia catalysts for diesel soot combustion. Catal. Today.

[11] Oi-Uchisawa J., Wang S., Nanba T., Ohi A., Obuchi A. (2003). Improvement of Pt catalyst for soot oxidation using mixed oxide as a support. Appl. Catal. B Environ..

[12] Wei Y., Zhao Z., Li T., Liu J., Duan A., Jiang G. (2014). The novel catalysts of truncated polyhedron Pt nanoparticles supported on three-dimensionally ordered macroporous oxides (Mn, Fe, Co, Ni, Cu) with nanoporous walls for soot combustion. Appl. Catal. B Environ..

[13] Nascimento L.F., Martins R.F., Silva R.F., Filho P.C.D.S., Serra O.A. (2014). Ru-doped ceria-zirconia mixed oxides catalyze soot combustion. Reac. Kinet. Mech. Cat..

[14] Jin B., Wei Y., Zhao Z., Liu J., Li Y., Li R., Duan A., Jiang G. (2017). Three-dimensionally ordered macroporous CeO_2_/Al_2_O_3_-supported Au nanoparticle catalysts: Effects of CeO_2_ nanolayers on catalytic activity in soot oxidation. Chin. J. Catal..

[15] Liu J., Zhao Z., Lan J., Xu C., Duan A., Jiang G., Wang X. (2009). Catalytic Combustion of Soot over the Highly Active (La_0.9_K_0.1_CoO_3_) x/nmCeO_2_ Catalysts. J. Phys. Chem. C.

[16] Hernández W.Y., Tsampas M.N., Zhao C., Boreave A., Bosselet F., Vernoux P. (2015). La/Sr-based perovskites as soot oxidation catalysts for Gasoline Particulate Filters. Catal. Today.

[17] Weng D., Li J., Wu X., Si Z. (2011). Modification of CeO_2_-ZrO_2_ catalyst by potassium for NOx-assisted soot oxidation. J. Environ. Sci..

[18] López-Suárez F.E., Bueno-López A., Illán-Gómez M.J. (2008). Cu/Al_2_O_3_ catalysts for soot oxidation: Copper loading effect. Appl. Catal. B Environ..

[19] Guilhaume N., Bassou B., Bergeret G., Bianchi D., Bosselet F., Jouguet B., Mirodatos C. (2012). In situ investigation of Diesel soot combustion over an AgMnOx catalyst. Appl. Catal. B Environ..

[20] Bueno-López A., Moulijn J.A., Krishna K., Makkee M. (2007). Potential rare earth modified CeO_2_ catalysts for soot oxidation I. Characterisation and catalytic activity with O_2_. Appl. Catal. B Environ..

[21] Rangaswamy A., Sudarsanam P., Reddy B.M. (2015). Rare earth metal doped CeO_2_-based catalytic materials for diesel soot oxidation at lower temperatures. J. Rare Earths.

[22] Bueno-López A., Krishna K., Van Der Linden B., Mul G., Moulijn J., Makkee M. (2007). On the mechanism of model diesel soot-O_2_ reaction catalysed A TAP study with isotopic O_2_. Catal. Today.

[23] Kustov A.L., Ricciardi F., Makkee M. (2009). NOx Storage and High Temperature Soot Oxidation on Pt–Sr/ZrO_2_ Catalyst. Top. Catal..

[24] Nascimento L.F., Martins R.F., Serra O.A. (2014). Catalytic combustion of soot over Ru-doped mixed oxides catalysts. J. Rare Earths.

[25] Wei Y., Zhao Z., Jiao J., Liu J., Duan A., Jiang G. (2014). Preparation of ultrafine Ce-based oxide nanoparticles and their catalytic performances for diesel soot combustion. J. Rare Earths.

[26] Liu S., Wu X.D., Weng D., Ran R. (2015). Ceria-based catalysts for soot oxidation: A review. J. Rare Earths.

[27] Liu S., Wu X., Liu W., Chen W., Ran R., Li M., Weng D. (2016). Soot oxidation over CeO_2_ and Ag/CeO_2_: Factors determining the catalyst activity and stability during reaction. J. Catal..

[28] Lin F., Delmelle R., Vinodkumar T., Reddy B.M., Wokaun A., Alxneit I. (2015). Correlation between the structural characteristics, oxygen storage capacities and catalytic activities of dual-phase Zn-modified ceria nanocrystals. Catal. Sci. Technol..

[29] Zhou L., Li X., Yao Z., Chen Z., Hong M., Zhu R., Liang Y. (2016). Transition-Metal Doped Ceria Microspheres with Nanoporous Structures for CO Oxidation. Sci. Rep..

[30] Yang Z., Hu W., Zhang N., Li Y., Liao Y. (2019). Facile synthesis of ceria–zirconia solid solutions with cubic–tetragonal interfaces and their enhanced catalytic performance in diesel soot oxidation. J. Catal..

[31] Shan W., Ma N., Yang J., Dong X., Liu C., Wei L. (2010). Catalytic oxidation of soot particulates over MnOx-CeO_2_ oxides prepared by complexation-combustion method. J. Nat. Gas. Chem..

[32] Venkataswamy P., Jampaiah D., Rao K.N., Reddy B.M. (2014). Nanostructured Ce_0.7_Mn_0.3_O_2-δ_ and Ce_0.7_Fe_0.3_O_2-δ_ solid solutions for diesel soot oxidation. Appl. Catal. A Gener..

[33] Grabchenko M.V., Mamontov G.V., Zaikovskii V.I., La Parola V., Liotta L.F., Vodyankina O.V. (2019). The role of metal–support interaction in Ag/CeO_2_ catalysts for CO and soot oxidation. Appl. Catal. B Environ..

[34] Grabchenko M.V., Mikheeva N.N., Mamontov G.V., Salaev M.A., Liotta L.F., Vodyankina O. (2018). Ag/CeO_2_ Composites for Catalytic Abatement of CO, Soot and VOCs. Catalysts.

[35] Neeft J.P.A., Makkee M., Moulijn J.A. (1996). Metal oxides as catalysts for the oxidation of soot. Chem. Eng. J..

[36] Araújo A.J.M., Silva V.D., Sousa A.R.O., Grilo J.P.F., Simões T.A., Macedo D.A., Nascimento R.M., Paskocimas C.A. (2018). Battery-like behavior of Ni-ceria based systems: Synthesis, surface defects and electrochemical assessment. Ceram. Int..

[37] Kumar S., Kim Y.J., Koo B.H., Lee C.G. (2010). Structural and magnetic properties of Ni doped CeO_2_ nanoparticles. J. Nanosci. Nanotechnol..

[38] Wu G., Wang X., Chen B., Li J., Zhao N., Wei W., Sun Y. (2007). Fluorine-modified mesoporous Mg-Al mixed oxides: Mild and stable base catalysts for O-methylation of phenol with dimethyl carbonate. Appl. Catal. A Gene.

[39] Leofanti G., Padovan M., Tozzola G., Venturelli B. (1998). Surface area and pore texture of catalysts. Catal. Today.

[40] Atzori L., Cutrufello M.G., Meloni D., Cannas C., Gazzoli D., Monaci R., Sini M.F., Rombi E. (2018). Highly active NiO-CeO_2_ catalysts for synthetic natural gas production by CO_2_ methanation. Catal. Today.

[41] Le T.A., Kim T.W., Lee S.H., Park E.D. (2018). Effects of Na content in Na/Ni/SiO_2_ and Na/Ni/CeO_2_ catalysts for CO and CO_2_ methanation. Catal. Today.

[42] Xu S., Yan X., Wang X. (2006). Catalytic performances of NiO–CeO_2_ for the reforming of methane with CO_2_ and O_2_. Fuel.

[43] Van Setten B.A.A.L., Makkee M., Moulijn J.A. (2007). Science and technology of catalytic diesel particulate filters. Catal. Rev. Sci. Eng..

[44] Stanmore B.R., Brilhac J.F., Gilot P. (2001). The oxidation of soot: A review of experiments, mechanisms and models. Carbon.

[45] Bueno-López A., Krishna K., Makkee M., Moulijn J.A. (2005). Enhanced soot oxidation by lattice oxygen via La3+ doped CeO_2_. J. Catal..

[46] Guillén-hurtado N., Bueno-lópez A., García-garcía A. (2012). Catalytic performances of ceria and ceria-zirconia materials for the combustion of diesel soot under NOx/O_2_ and O_2_. Importance of the cerium precursor salt. Appl. Catal. A Gene.

[47] Wen Z., Duan X., Hu M., Cao Y., Ye L., Jiang L., Yuan Y. (2018). Efficient low-temperature soot combustion by bimetallic Ag-Cu/SBA-15 catalysts. J. Environ. Sci..

[48] Hu Z., Qiu S., You Y., Guo Y., Guo Y., Wang L., Zhan W., Lu G. (2018). Hydrothermal synthesis of NiCeOx nanosheets and its application to the total oxidation of propane. Appl. Catal. B Environ..

[49] Liu Y.M., Wang L.C., Chen M., Xu J., Cao Y., He H.Y., Fan K.N. (2009). Highly Selective Ce–Ni–O Catalysts for Efficient Low Temperature Oxidative Dehydrogenation of Propane. Catal. Lett..

[50] Neeft J.P.A., Nijhuis T.X., Smakman E., Makkee M., Moulijn J.A. (1997). Kinetics of the oxidation of diesel soot. Fuel.

