# Supplementary material for: Catalytic Soot Oxidation Activity of NiO–CeO2 Catalysts Prepared by a Coprecipitation Method: Influence of the Preparation pH on the Catalytic Performance
Source: Materials (Basel). 2019 Oct 21;12(20):3436. doi: 10.3390/ma12203436 (PMC6829405; doi:10.3390/ma12203436)
Supplement: Supplementary file 1 [file materials-12-03436-s001.pdf]

materials

MDPI

Article

Catalytic Soot Oxidation Activity of NiO-CeO_2 Catalysts Prepared by a Coprecipitation Method: Influence of the Preparation pH on the Catalytic Performance

Amar Bendieb Aberkane [1,*], María Pilar Yeste [2,*], Djazi Fayçal [1], Daniel Goma [2] and Miguel Ángel Cauqui [2]

1 Laboratoire de recherche sur la physico-chimie des surfaces et interfaces (LRPCSI), Département de Pétrochimie & Génie des Procédés, Faculté de Technologie, Université 20 Août 1955-Skikda, BP 26, Route d'El Hadaiek-Skikda 21000, Algérie

2 Department of Material Science, Metallurgical Engineering and Inorganic Chemistry, Faculty of Sciences, University of Cadiz, E-11510, Puerto Real, 11003 Cadiz, Spain.

* Correspondence: a.bendiebaberkane@univ-skikda.dz (A.B.A.); pili.yeste@uca.es (M.P.Y.)

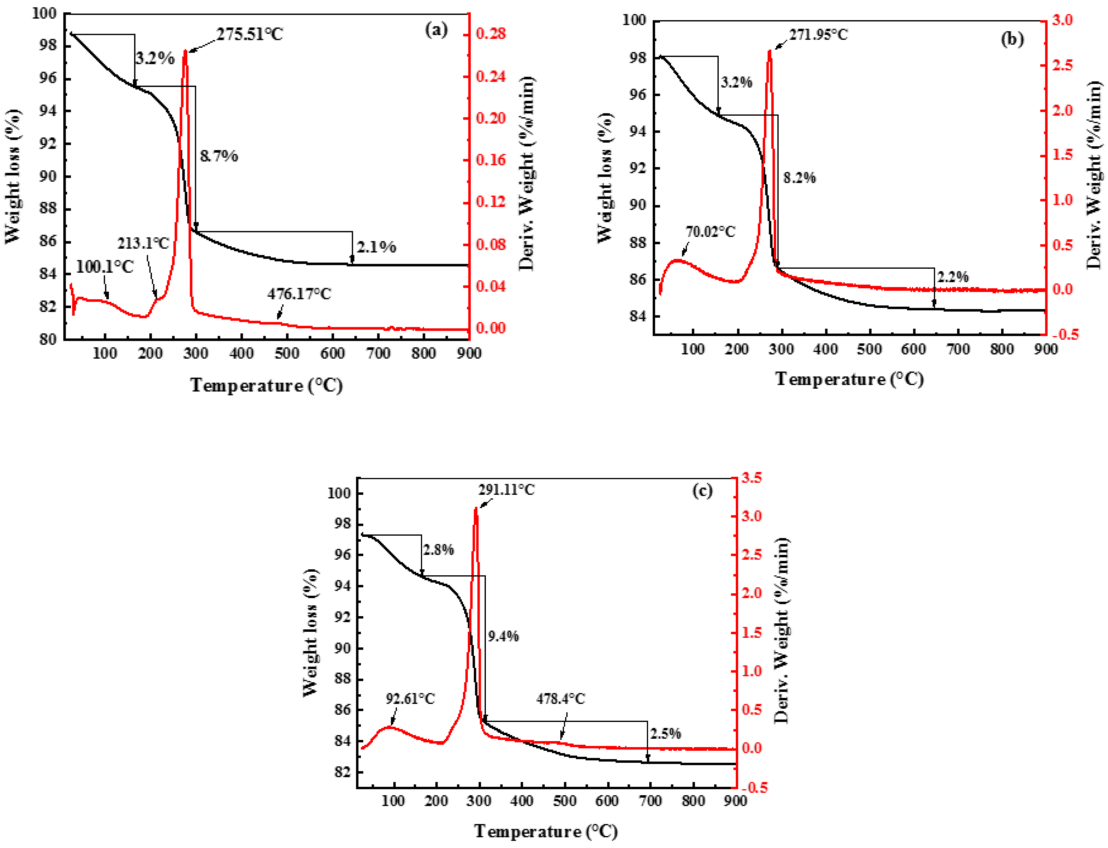

Figure S1. TGA Profile of Ni-Ce-pH catalysts: (**a**) Ni-Ce-8; (**b**) Ni-Ce-9 and (**c**) Ni-Ce-10.

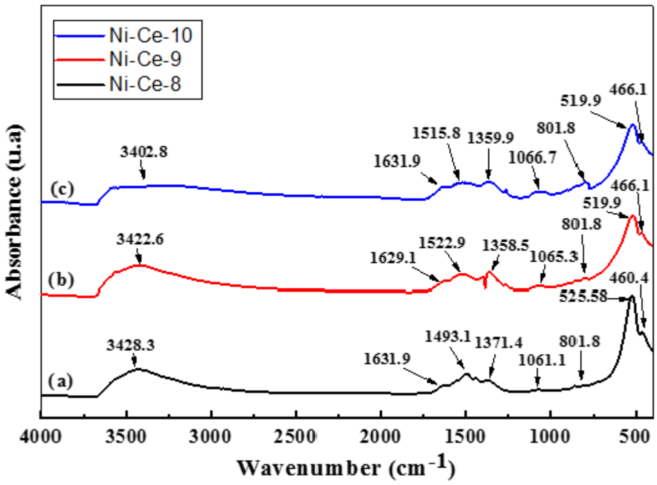

Figure S2. FTIR Spectrum of Ni-Ce-pH catalysts before calcination: (**a**) Ni-Ce-8; (**b**) Ni-Ce-9 and (**c**) Ni-Ce-10.

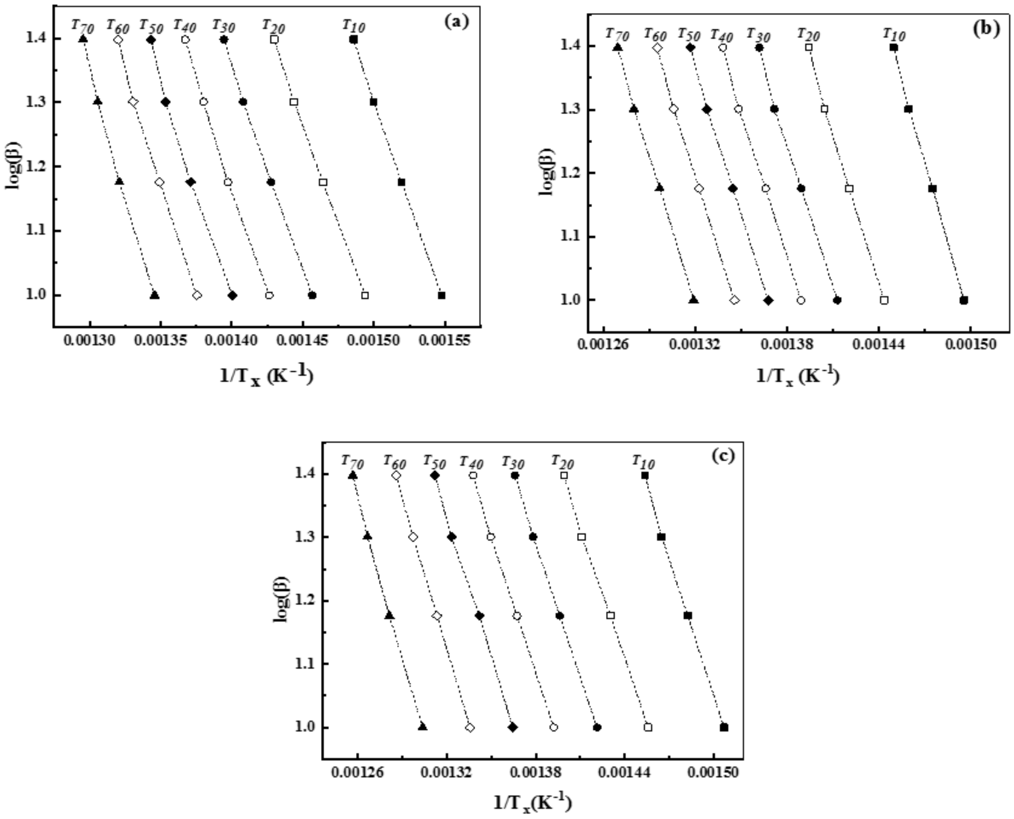

Figure S3. Ozawa plots at different soot conversion over (**a**) Ni-Ce-8; (**b**) Ni-Ce-9 and (**c**) Ni-Ce-10 at different soot conversion levels (x%), with (β) different heating applied during the soot oxidation, and T_x (temperature at 'x%' conversion). Reaction conditions: TGA, catalyst: soot—20:1 (w:w), contact: tight, β = 10, 15, 20 and 25 °C/min, air/N_2 flow = 100mL/min (air/N_2 : 60%/40%).

© 2019 by the authors. Submitted for possible open access publication under the terms and conditions of the Creative Commons Attribution (CC BY) license (http://creativecommons.org/licenses/by/4.0/).
